# Nutritional potential of invasive *Rugulopteryx okamurae*, of *Gelidium corneum* industrial by-product, and of five native Atlantic macroalgae as feedstocks for alternative protein production

**DOI:** 10.3389/fnut.2026.1959089

**Published:** 2026-09-28

**Authors:** Kaifeng Zhao, Carla Cameselle, Celia Costas, Weronika Orzechowska, Łukasz Woźniak, Beatriz Rodríguez-Martínez, M. Teresa Cesário, Jesus Simal-Gandara, Paz Otero

**Affiliations:** 1 Department of Analytical and Food Chemistry, Universidade de Vigo, Faculty of Science, Ourense, Spain; 2 Instituto de Agroecoloxía e Alimentación (IAA), Universidade de Vigo, Campus Auga, Ourense, Spain; 3 Department of Food Safety and Chemical Analysis, Institute of Agricultural and Food Biotechnology – State Research Institute, Warsaw, Poland; 4 Departamento de Enxeñaría Química, Facultade de Ciencias, Universidade de Vigo, Ourense, Spain; 5 Department of Bioengineering, iBB-Institute for Bioengineering and Biosciences, Instituto Superior Técnico, Universidade de Lisboa, Lisbon, Portugal; 6 Associate Laboratory i4HB—Institute for Health and Bioeconomy, Instituto Superior Tecnico, Universidade de Lisboa, Lisbon, Portugal; 7 Agricultural and Food Research Group (AA1), Galicia Sur Health Research Institute (IIS Galicia Sur), SERGAS-UVIGO, Vigo, Spain; 8 CISPAC, Fontan Building, City of Culture, Santiago de Compostela, Spain

**Keywords:** *Cystoseira usneoides*, fatty acids, full nutritional characterization, phenolic compounds, protein quality

## Abstract

Macroalgae are increasingly considered sustainable resources for protein-oriented food and bioprocessing applications due to their rapid growth, wide availability, low freshwater and fertilizer requirements, and potential as natural sources of proteins and bioactive compounds. However, integrated assessments comparing native, invasive, and industrial side-stream macroalgal biomasses under the same nutritional and safety framework remain scarce, limiting the identification of suitable candidates for protein-oriented valorization. In this context, this study assessed seven Atlantic macroalgal biomasses from Spanish and Portuguese sources: the invasive brown seaweed *Rugulopteryx okamurae* (*R. okamurae*), the underutilized *Cystoseira usneoides* (*C. usneoides*), an industrial *Gelidium corneum* (*G. corneum*) by-product, and four commonly studied species, *Saccharina latissima* (*S. latissima*), *Porphyra umbilicalis* (*P. umbilicalis*), *Gracilaria gracilis* (*G. gracilis*), and *Ulva rigida* (*U. rigida*). A common analytical framework was applied to determine proximate composition, carbon-to-nitrogen ratio, amino acid and fatty acid profiles, nutritional indices, mineral and phenolic contents, heavy metals and *in vitro* cytotoxicity. The results showed that the red algae *P. umbilicalis* and *G. gracilis* exhibited the highest protein content among the evaluated macroalgae, with values of 30.78 and 22.69%, respectively, together with relatively low C/N ratio indicating nitrogen-rich compositions. Leucine, isoleucine, and phenylalanine were the predominant essential amino acids, whereas the *G. corneum* by-product displayed the highest total essential amino acid content (44.8 g/100 g protein) and essential amino acid index (141%). Most biomasses exceeded an essential amino acid index of 90%, supporting their nutritional relevance. Fatty acid analysis revealed unusually high monounsaturated fatty acids levels in *R. okamurae* and *C. usneoides*, with *R. okamurae* being the only brown alga containing both EPA and DHA. Moreover, *in vitro* cytotoxicity assays confirmed satisfactory biocompatibility and safety for most algal extracts. Overall, *G. gracilis*, *P. umbilicalis* and the *G. corneum* by-product emerged as the most promising candidates for protein-oriented valorization, providing a basis for the development of alternative protein ingredients and higher-value uses of macroalgal resources.

## Graphical abstract

**Figure:**
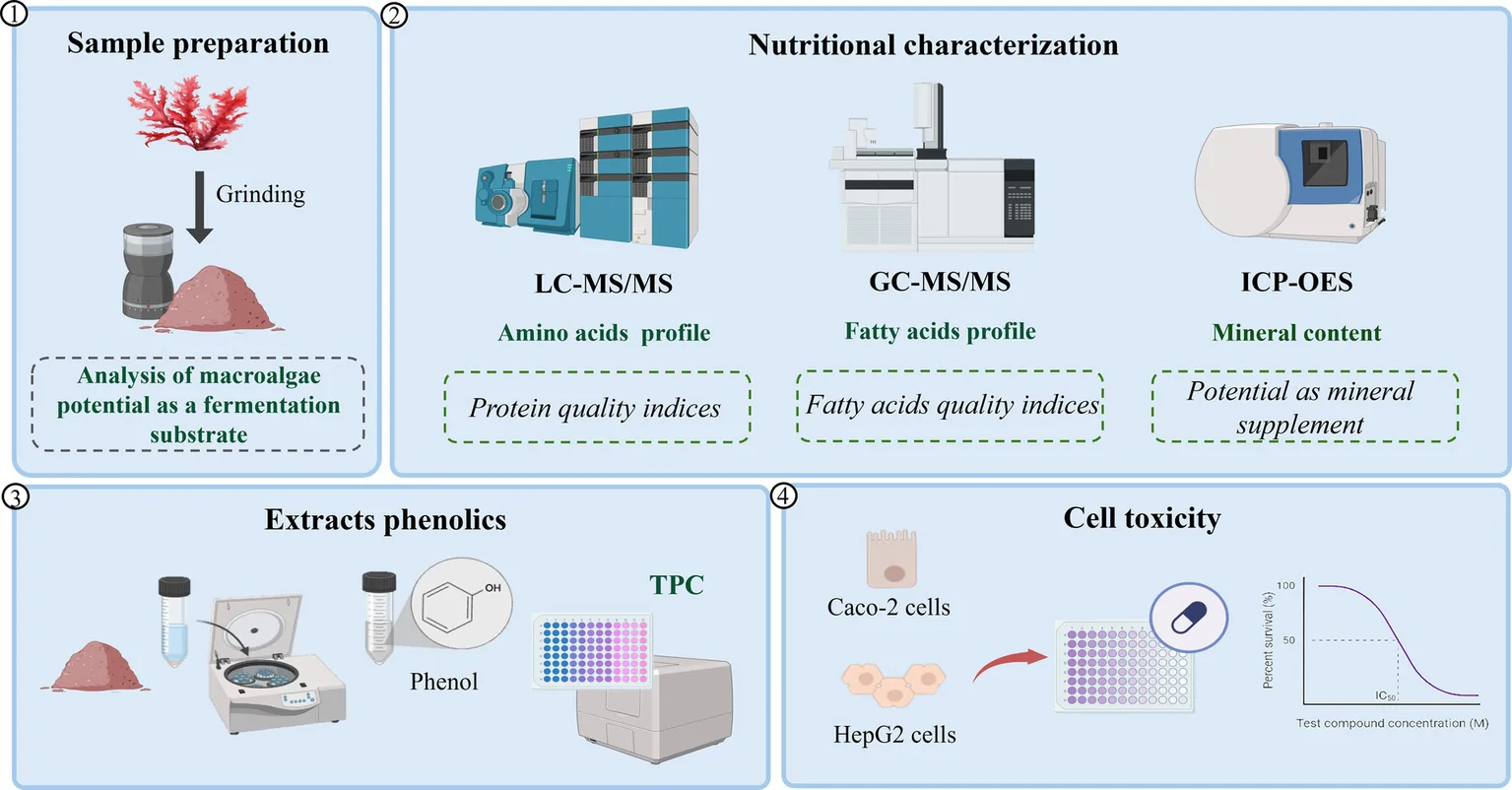

## 1 Introduction

Against the backdrop of the global pursuit of sustainable social and economic development, marine resources are increasingly recognized as strategic sources of nutrients and bio-based compounds. According to a report by the Food and Agriculture Organization of the United Nations (FAO), the total global production of fisheries and aquaculture reached 223.2 million tons in 2022, while the output of algae was 37.8 million tons, of which only 0.8% comes from Europe (1). However, the increasing pressure on fisheries and the overexploitation of marine resources in several regions highlight the urgent need for more sustainable alternatives for protein supply. Against this backdrop, macroalgae have emerged as promising renewable biomasses owing to their high productivity, rapid growth, and lack of reliance on fresh water and chemical fertilizers (2).

Macroalgae, mainly classified as red algae (Rhodophyta), brown algae (Phaeophyceae), and green algae (Chlorophyta), are widely distributed in the coastal waters around the world and represent natural sources of bioactive compounds (3). Their composition may include carbohydrates (accounting for up to 60% of their dry weight), high-quality protein (including all essential amino acids), lipids (especially unsaturated fatty acids), carotenoids, polyphenols, and other functional components (4). In addition, macroalgae are rich in a variety of micronutrients, such as vitamins A, C, E, complex B, as well as minerals like calcium, magnesium, iron, potassium, phosphorus, and sodium. Their ash content can reach up to 40% of dry weight, and their mineral content is even 10 to 100 times higher than those found in terrestrial vegetables (5, 6). Due to their high bioavailability and nutritional richness, macroalgae have been widely studied in multiple fields, such as nutritional supplements, health foods, and medicinal products (4, 7).

Recent studies have also shown that production systems and cultivation conditions can significantly influence the growth performance, proximate composition, amino acid profile, and fatty acid composition of edible macroalgae, reinforcing the need for species- and source-specific nutritional characterization (8). Previous works have described the nutritional composition, chemical characteristics, and biological activities of several edible macroalgae, including the red algae *P. umbilicalis* and *G. gracilis*, as well as their applications in industrial processing and regulation of human intestinal flora (9, 10). The brown alga *S. latissima* is also widely studied and cultivated along the Atlantic coast, being recognized as a source of various compounds with antioxidant, antibacterial, and antihypertensive effects (11), as well as one of the main sources of commercial alginate (12). However, information remains more limited for other Atlantic brown algae, such as *C. usneoides* and *R. okamurae* and for industrial side streams derived from *G. corneum* processing. *C. usneoides* is an underutilized native brown alga, while *R. okamurae* is an invasive species that has rapidly expanded along southern Spain and nearby coastal areas in recent years. Despite its ecological relevance, its nutritional potential, valorization routes and food safety implications remain insufficiently explored. Similarly, although *G. corneum* is industrially exploited for agar extraction, the resulting processing by-products remain poorly characterized as potential sources of nutrients and functional compounds. The use of macroalgae as food or food-related ingredients also requires careful safety assessment. In Europe, the commercialization of new types of macroalgae into the European Union (EU) market is regulated by Novel Food Regulation (EU) 2015/2283 (13). Furthermore, the EU Recommendation (EU) 2018/464 required member states to monitor the content of heavy metals, including arsenic (As), cadmium (Cd), lead (Pb), and mercury (Hg), in seaweeds, halophytes and alginate-based products in order to assess the long-term dietary exposure and potential risks (14). This concern is supported by recent global evidence showing that seaweeds may bioaccumulate potentially toxic elements as a result of both natural and anthropogenic factors, with relevant implications for food safety, public health and regulatory monitoring (15). In this regard, the European Food Safety Authority (EFSA) has identified inorganic arsenic (iAs) as an important dietary risk associated with macroalgae, and determines that the BMDL_01_ value is 0.3–8.0 μg·kg^−1^ in the risk characterization of iAs (16). Moreover, member states such as France also have recommended limits for iAs in condiments/seaweed and vegetable categories (17). Although heavy metals, As species and iodine (I) have been monitored in species such as *S. latissima* and *P. umbilicalis* under different geographical and sampling conditions, and reported that iAs species in seaweed showed a risk of low As intake (18). However, at present, the regulations have not stipulated specific limits for species such as *R. okamurae*, *C. usneoides* and *G. corneum*. Therefore, the evaluation of potentially toxic elements is essential to determine their suitability for food, feed or bioprocessing applications.

In recent years, the assessment of *in vitro* biological activity and cytotoxicity of macroalgae extracts has gradually increased. However, most existing works have focused on the antioxidant, anti-inflammatory, or anti-tumor activities of algal extracts or fractions, commonly using experimental models based on immune, tumor or skin cell lines (19). For instance, the diterpenoids of *R. okamurae* have been demonstrated to have anti-inflammatory effects in RAW264.7 and Bv.2 cells (20), while the brown algae polysaccharides of *S. latissima* have shown certain cytotoxicity in cells models such as HepG2 (21). However, studies that conduct systematic dose–response toxicological evaluations based on human intestinal epithelial cells (Caco-2) and hepatocyte lines (HepG2), combined with the analysis of pollutants such as As, remain scarce. Such an integrated approach is necessary to better estimate the potential risks associated with dietary exposure and to support the safe valorization of macroalgal resources.

Although the nutritional and functional potential of several macroalgae has been previously reported, their composition is highly influenced by species, harvesting season, geographical origin and cultivation or collection conditions. Consequently, comparative studies performed under a common analytical framework are still needed to identify the most suitable macroalgal feedstocks for protein recovery and bioproduct development. This gap is even more evident for less-explored biomasses and industrial side streams, since approximately 20% of the biomass generated during algae processing is still underutilized. Although it is often treated as a biofuel, it is still rich in potential functional components that can be used to develop alternative proteins, feed, or biomaterials (22). In this context, recent food reformulation studies have demonstrated the potential of seaweed-containing formulations to reduce dependence on conventional marine resources while maintaining or improving nutritional and fatty acid profiles, highlighting the relevance of validated compositional data for the development of sustainable protein-oriented food products (23).

Based on this rationale, the present study hypothesizes that invasive, underutilized and industrial by-product macroalgal biomasses from the Atlantic coast may represent nutritionally valuable and sufficiently safe feedstocks for future fermentation-based alternative protein production. To test this hypothesis, seven macroalgal biomasses obtained from Spanish and Portuguese harvest, aquaculture and industrial sources were comparatively characterized under a common analytical framework. The study focused on three scarcely explored or underutilized resources: the invasive brown seaweed *R. okamurae*, the native brown seaweed *C. usneoides* and an industrial *G. corneum* by-product. Four more commonly studied species, namely *S. latissima* (brown), *P. umbilicalis* (red), *G. gracilis* (red) and *U. rigida* (green), were included as reference biomasses. Their proximate composition, carbon-to-nitrogen ratio, amino acid and fatty acid profiles, nutritional indices, mineral and phenolic contents, heavy metal levels and *in vitro* cytotoxicity were evaluated to identify the most promising candidates for protein-oriented valorization. The classification of the studied biomasses is shown in Figure 1.

**Figure 1:**
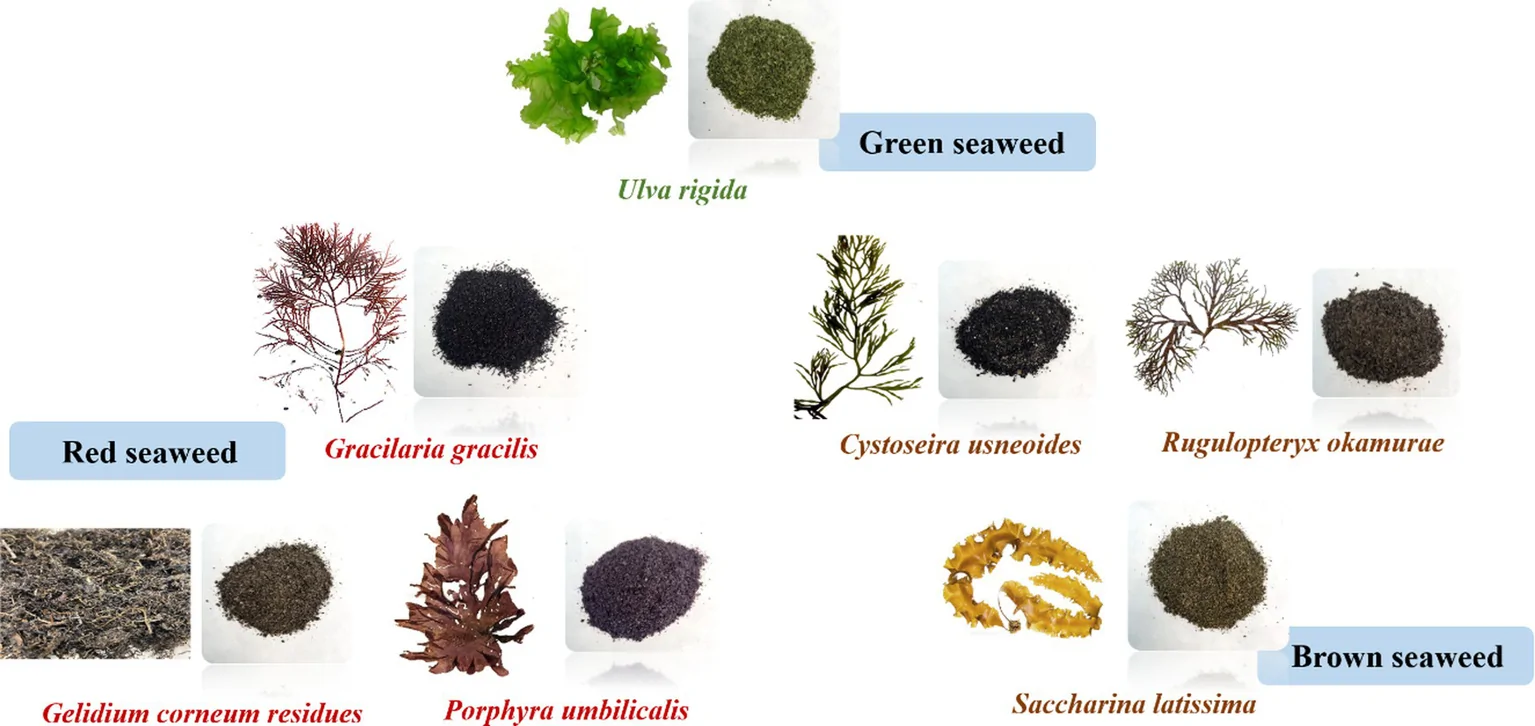
Macroalgae species included in this study.

## 2 Materials and methods

### 2.1 Chemicals and reagents

Six macroalgae species were obtained from Portuguese and Spanish harvesting or aquaculture companies: *S. latissima*, *U. rigida*, *G. gracilis*, *P. umbilicalis*, *R. okamurae* and *C. usneoides.* In addition, an industrial by-product derived from *G. corneum* processing was obtained from a phycocolloid extraction industry.

*U. rigida*, *G. gracilis,* and *S. latissima* were supplied by the Portuguese company ALGAplus Lda. (Ílhavo, Portugal). *U. rigida* and *G. gracilis* were produced in open land-based integrated multitrophic aquaculture (IMTA) systems located in the Ria de Aveiro coastal lagoon, whereas *S. latissima,* originally from the United Kingdom, was commercialized by the same company. The *G. corneum* by-product, produced after agar-agar extraction, was kindly supplied by Iberagar-Sociedade Luso-Espanhola de Colóides Marinhos S. A. (Coina, Portugal). This biomass was provided as a dry ground powder containing heterogeneous particle sizes ranging from approximately 1.3 mm × 1.9 mm to 71 μm × 90 μm or smaller. After harvesting and drying, the biomass undergoes a previous alkaline pre-treatment before agar extraction with boiling water. Thereafter, the hot solution is filtered to separate the dissolved agar from the solid seaweed residue. The residues are sun-dried and ground.

*P. umbilicalis* was supplied by the Galician company ALGAS ATLÁNTICAS ALGAMAR, S. L., as dried and ground biomass with a particle size of approximately 1.0 mm. This macroalga was manually harvested along the coast of Galicia and dried at low temperatures. The species *C. usneoides* and *R. okamurae* were supplied as dried, non-ground biomasses. Drying was carried out in a non-convective oven at 40 °C. *C. usneoides* was collected on the beach of the peninsula Tróia- Grândola-Portugal, while *R. okamurae* was manually harvested off the coast of isle of São Miguel- Açores - Portugal.

All reagents used in this study were of analytical or LC–MS grade and were purchased from commercial suppliers as specified below. Milli-Q water was produced using a Milli-Q purification system (Merck Millipore S. A. S, Molsheim, France) and was used throughout all experiments. Methanol (MeOH, LC–MS grade) and ethanol (EtOH) were obtained from Merck K GaA (Darmstadt, Germany) and were used for the extraction of macroalgae samples. Formic acid, glacial acetic acid, and acetic acid were also purchased from Merck KGaA (Darmstadt, Germany). Hydrochloric acid (HCl, 37%) and nitric acid (HNO_3_, 65%) used for sample preparation prior to nutrition compounds analysis were obtained from Sigma-Aldrich (St. Louis, MO, USA). Hydrogen peroxide (H_2_O_2_, 30%) was purchased from Merck KGaA (Darmstadt, Germany) and was used in heavy metal procedures during proximate analysis. Sodium chloride (NaCl) and sodium bicarbonate (NaHCO_3_) were supplied by Thermo Fisher Scientific (Waltham, MA, USA) and used for fatty acids analysis. The Folin–Ciocalteu reagent (Sigma-Aldrich, St. Louis, MO, USA), sodium carbonate (Na_2_CO_3_, Sigma-Aldrich, St. Louis, MO, USA), and gallic acid (calibration standard, Sigma-Aldrich, St. Louis, MO, USA) were used for the microplate-based colorimetric assay of total polyphenol content (TPC).

### 2.2 Macroalgae sample preparation

All macroalgal samples were processed in accordance with the following standardized procedures before analysis. Macroalgae were washed with seawater, centrifuged to remove excess water, and then dried in a forced air-tunnel at a set temperature of 25 °C until reaching a moisture content of 10–11%. After collection and drying, the samples were crushed evenly by a grinder and stored at room temperature, protected from light for subsequent experimental analysis. A total of 7 macroalgae samples and species were included in this study, as shown in Table 1.

**Table 1:** Detailed of macroalgae samples and species and their origin.

N°	Specie	Category	Sampling site	Biomass
1	*Ulva rigida (U. rigida)*	Green algae	Ria de Aveiro coastal lagoon (Portugal)	Macroalgae
2	*Saccharina latissima (S. latissima)*	Brown algae	United kingdom (UK)	Macroalgae
3	*Porphyra umbilicalis (P. umbilicalis)*	Red algae	Galician Coast (Spain)	Macroalgae
4	*Gelidium corneum (G. corneum)*	Red algae	Coast of S. Martinho do Porto (Portugal)	Industrial residue
5	*Cystoseira usneoides (C. usneoides)*	Brown algae	Beach of the peninsula Tróia, Grândola (Portugal)	Macroalgae
6	*Rugulopteryx okamurae (R. okamurae)*	Brown algae	Coast of isle of São Miguel, Açores (Portugal)	Macroalgae (Invasive)
7	*Gracilaria gracilis (G. gracilis)*	Red algae	Ria de Aveiro coastal lagoon (Portugal)	Macroalgae

### 2.3 Nutritional characterization

#### 2.3.1 Ash

The biomass was ground, sieved, and stored at −20 °C until subsequent analysis. Moisture content was determined by an oven-drying method based on AOAC Official Method 930.04 (Loss on Drying (Moisture) in Plants), with modification (24). Briefly, the samples were dried at 105 °C for 24 h until constant weight was achieved.

Ash content was determined based on AOAC Official Method 930.05 (Ash of Plants). Approximately 2–5 g of dried macroalgal biomass was pre-charred and subsequently incinerated in a muffle furnace at 550 °C for 4 h. After cooling in a desiccator, the remaining inorganic residue was weighed, and the ash content was expressed as a percentage of the original dry biomass.

#### 2.3.2 Total protein content and amino acid profile

The total protein content was determined by the combustion (Dumas) method following the procedure described by Miller (25) using a FISONS Carlo Erba EA1108 Elemental Analysis Unit. Samples were subjected to high-temperature combustion, and the resulting gases were passed through a copper reduction column, where nitrogen oxides were reduced to molecular nitrogen (N₂). Helium was used as the carrier gas. The gaseous products were subsequently separated using the gas chromatographic system integrated into the elemental analyzer and quantified using a thermal conductivity detector (TCD). Based on reported data for green, red, and brown macroalgae (26), an average nitrogen-to-protein conversion factor (N-Prot) of 4.12 was adopted to improve the accuracy of protein estimation, and protein content was expressed as g/100 g dry biomass.

The amino acid composition of the seven macroalgal biomasses was determined using an acid hydrolysis procedure based on AOAC Official Method 994.12, with modifications (24). 50 mg of sample was added to a test tube containing 1 mL of 6 N HCl and hydrolyzed at 112 °C for 22 h. Amino acid separation was performed using a C18 HPLC column (Phenomenex Luna, 150 mm × 2 mm × 3 μm). Amino acids composition in the macroalgae, including histidine (His), isoleucine (Ile), leucine (Leu), lysine (Lys), methionine (Met), phenylalanine (Phe), threonine (Thr), valine (Val), alanine (Ala), arginine (Arg), aspartic acid (Asp), cysteine (Cys), glutamic acid (Glu), glycine (Gly), proline (Pro), serine (Ser), and tyrosine (Tyr) were analyzed using an Agilent 1,260 Infinity series system (Agilent, Palo Alto, CA, USA) coupled with a triple quadrupole mass spectrometer (Triple Quad 3,500, AB SCIEX INSTRUMENTS, Foster City, CA, USA). The system was equipped with an electrospray ionization (ESI) source using Turbo V™ technology, operated in both positive and negative ion modes, with nitrogen used as the nebulizing and collision gas. The mobile phase consisted of solvent A (water with 0.1% acetic acid) and solvent B (ACN with 0.1% acetic acid). The injection volume was 10 μL, the column temperature was maintained at 30 °C, and the flow rate was set at 0.3 mL/min. Data were processed using Analyst 1.6.2 Software (AB Sciex, Foster City, CA, USA).

It should be noted that the official acid hydrolysis method of AOAC may lead to the degradation of some amino acids. Specifically, under acidic conditions, tryptophan is destroyed and cysteine is prone to oxidation, which may lead to an underestimation of its measured value.

#### 2.3.3 Protein quality indices: chemical score, essential amino acid index, and protein digestibility-corrected amino acid score

After quantified the individual amino acids of the samples, the chemical score (CS) of essential amino acids (EAAs) was calculated by comparing the amino acid composition of each sample with the adult indispensable amino acid requirement pattern recommended by the WHO/FAO/UNU (27). The calculation employed the following Equation 1:

$$\mathrm{CS}\;(\%)=\frac{g\;\mathrm{EAA}\;\text{in tested protein}}{g\;\mathrm{EAA}\;\text{in pattern protein}}\times 100\;$$ (1)

The essential amino acid index (EAAI) was determined according to the Equation 2 presented by (28):

$$\text{EAAI}\;(\%)={\left(\prod_{i=1}^{n}(\frac{a_{i}}{A_{i}})\right)}^{1/n}\times 100\;$$ (2)

Where the a_i_ means the i-th EAA in the samples, and A_i_ represents the corresponding protein pattern according to the FAO/WHO reference. n is the number of amino acids considered for the calculation (the pair methionine-cysteine count as 1).

The Protein Digestibility-Corrected Amino Acid Score (PDCAAS) was used to evaluate the protein quality based on both amino acid content and digestibility, which is represented following Equation 3:

$$\text{PDCAAS}\;(\%)=\frac{\begin{array}{l} \mathrm{mg}\;\text{of limiting amino}\;\\ \text{acid}\;\mathrm{per}\;g\;\text{of test protein} \end{array}}{\begin{array}{l} \mathrm{mg}\;\text{of same amino acid}\;\\ \mathrm{per}\;g\;\text{of reference protein} \end{array}}\times \mathrm{DF}\times 100\;$$ (3)

Where the DF represents the digestibility factor, which was assumed to be 0.8 based on literature values for macroalgae proteins (29).

#### 2.3.4 Total lipid content and fatty acids profile

The total lipid content was determined gravimetrically following the Folch method with modifications (30). Briefly, 1 g of dry biomass was extracted with 25 mL of CHCl_3_: MeOH (2:1 v/v) and vortexed. After centrifugation at 7000 rpm for 5 min, the supernatant was washed with Milli-Q water to remove polar impurities. The tube was vigorously vortexed and dried under nitrogen. Total lipid content was determined by comparing the weight of the dried extract with the original sample weight and expressed as g/100 g of dry biomass.

The identification and quantification of fatty acids (FAs) were carried out following a modified version of the method described by Otero (31). Briefly, 1 mL of toluene and 2 mL of 1% H₂SO₄ in MeOH were added to the total lipid extract, and the samples were incubated overnight in a water bath at 50 °C. Subsequently, 5 mL of 5% NaCl solution was added, followed by two extractions with 5 mL of n-hexane. The phases were separated, and the n-hexane phase containing the lipids was collected. Then, 4 mL of 2% NaHCO_3_ solution was added to the lipid phase. The resulting sample was evaporated and resuspended in 1 mL of n-hexane for GC–MS analysis. An Agilent HP-5MS UI capillary column (30 m × 0.25 mm × 0.25 μm), a GC4513A autoinjector with electronic pressure control, and a 5975C triple-axis mass spectrometer detector were used. The injection volume was 1 μL in split mode. The injector temperature was set at 260 °C, and the ion source and MS interface temperatures were 230 °C and 280 °C, respectively. The oven temperature program started at 50 °C, increased to 210 °C at a rate of 20 °C/min, and was held for 18 min. Then, the temperature was raised to 230 °C and maintained for another 13 min. Data were acquired in full scan mode from 40 to 500 m/z.

#### 2.3.5 Fatty acids quality indices

The sum and percentage of saturated fatty acids (SFAs), monounsaturated fatty acids (MUFA) and polyunsaturated fatty acids (PUFA) are shown in Table 2.

**Table 2:** Fatty acids composition of macroalgae (mg/kg).

Fatty acid/category	Fatty acid name	*U. rigida*	*S. latissima*	*P. umbilicalis*	*G. corneum*	*C. usneoides*	*R. okamurae*	*G. gracilis*
FA	Total fatty acids(mg/kg)	3170.00 ± 115.48^c^	4854.11 ± 596.59^bc^	4874.62 ± 844.32^bc^	5246.47 ± 1039.30^abc^	6317.29 ± 2286.16^ab^	6974.26 ± 1089.32^a^	314.16 ± 26.44^d^
SFA	Saturated fatty acids	3046.54 ± 109.81^b^	4383.83 ± 584.35^ab^	3978.54 ± 743.08^ab^	4847.26 ± 838.17^a^	5172.55 ± 1699.81^a^	5458.37 ± 850.65^a^	295.44 ± 25.59^c^
C6:0	Caproic acid	55.94 ± 1.05^c^	70.36 ± 7.82^ab^	65.86 ± 6.63^b^	74.17 ± 1.44^ab^	78.13 ± 9.07^a^	73.49 ± 5.03^ab^	-
C8:0	Caprylic acid	-	-	-	-	-	-	-
C10:0	Capric acid	-	-	-	-	-	-	-
C11:0	Hendecanoic acid	-	-	-	-	-	-	-
C12:0	Lauric acid	12.61 ± 1.16	15.93 ± 2.84	-	-	-	-	-
C13:0	Tridecylic acid	-	-	-	-	-	-	-
C14:0	Myristic acid	43.39 ± 1.40^cd^	237.48 ± 34.73^b^	41.26 ± 5.49^cd^	123.01 ± 77.65^bcd^	98.20 ± 46.14^bc^	469.02 ± 122.40^a^	6.88 ± 0.30^d^
C15:0	Pentadecanoic acid	-	27.02 ± 3.56	-	-	-	67.83 ± 10.28	-
C16:0	Palmitic acid	1127.49 ± 55.00^b^	2049.68 ± 290.44^ab^	2245.59 ± 293.20^a^	2526.03 ± 444.91^a^	2565.55 ± 1192.25^a^	2845.58 ± 530.13^a^	76.84 ± 5.98^c^
C17:0	Margaric acid	14.25 ± 1.25	22.18 ± 4.31	-	-	-	-	-
C18:0	Stearic acid	1792.86 ± 56.21^b^	1961.17 ± 254.44^ab^	1625.83 ± 346.59^b^	2124.04 ± 336.63^ab^	2430.66 ± 371.69^a^	1943.99 ± 188.61^ab^	211.73 ± 20.08^c^
C21:0	Heneicosanoic acid	-	-	-	-	-	-	-
C23:0	Tricosanoic acid	-	-	-	-	-	-	-
C24:0	Lignoceric acid	-	-	-	-	-	58.45 ± 9.05	-
MUFA	Monounsaturated fatty acids	91.05 ± 7.09^c^	305.26 ± 20.98^bc^	303.85 ± 32.97^bc^	306.61 ± 193.65^c^	778.09 ± 414.17^ab^	766.20 ± 160.06^a^	5.70 ± 3.37^c^
C14:1n5	Myristoleic acid	-	-	47.82 ± 2.67^a^	61.42 ± 5.92^a^	50.21 ± 19.63^a^	53.24 ± 6.24^a^	5.70 ± 3.37^b^
C15:1n5	cis-10-pentadecenoic acid	8.44 ± 0.85	9.00 ± 0.87	-	-	-	-	-
C16:1n7	Palmitoleic acid	43.80 ± 3.54^b^	92.90 ± 10.24^b^	103.37 ± 5.52^b^	57.21 ± 59.54^b^	172.32 ± 148.15^ab^	245.14 ± 69.91^a^	-
C17:1n7	cis-10-heptadecanoic acid	-	-	-	-	-	-	-
C18:1n9 trans	Elaidic acid	-	-	-	-	-	-	-
C18:1n9 cis	Oleic acid	22.59 ± 1.73^b^	194.20 ± 11.63^ab^	53.26 ± 6.13^b^	187.98 ± 110.14^ab^	509.72 ± 40.94^a^	417.82 ± 88.72^a^	-
C20:1n9	cis-11-eicosenoic acid	16.22 ± 2.56^b^	-	99.38 ± 21.35^a^	-	45.85 ± 27.34^b^	33.98 ± 5.86^b^	-
C24:1n9	Nervonic acid	-	9.16 ± 1.02	-	-	-	16.02 ± 3.63	-
PUFA	Polyunsaturated fatty acids	32.41 ± 0.60^d^	165.02 ± 15.12^cd^	502.23 ± 182.94^b^	93.61 ± 18.05^cd^	266.44 ± 202.20^c^	749.70 ± 110.36^a^	13.01 ± 1.28^d^
C18:2n6 cis	Linoleic acid	-	57.88 ± 5.71^b^	42.04 ± 7.55^b^	47.05 ± 4.79^b^	119.90 ± 61.80^a^	62.85 ± 11.58^b^	-
C18:3n6	γ-Linolenic acid	-	-	-	-	-	22.90 ± 2.23	-
C20:0 + C18:3n3	Arachidic acid + Linolenic acid	32.41 ± 0.60^c^	52.43 ± 2.29^bc^	42.04 ± 12.71^bc^	45.56 ± 13.58^bc^	66.03 ± 23.22^b^	154.35 ± 21.54^a^	-
C20:2n6	cis-11,14-eicosadienoic acid	-	54.71 ± 7.97^a^	52.64 ± 8.53^ab^	-	36.19 ± 13.45^b^	16.04 ± 0.30^c^	-
C20:3n6	Dihomo-γ-linolenic Acid (DGLA)	-	-	29.32 ± 3.67	-	-	211.91 ± 40.47	-
C22:0 + C20:3n3 + C22:1n9	Behenic acid + cis-11,14,17-eicosatrienoic acid + Erucic acid	-	-	95.25 ± 17.82	-	72.71 ± 4.15	-	-
C20:4n6	Arachidonic acid	-	-	18.36 ± 2.67	-	-	-	13.01 ± 1.28
C20:5n3	Timnodonic acid (EPA)	-	-	311.97 ± 67.93^a^	-	71.83 ± 4.59^b^	213.41 ± 40.24^ab^	-
C22:2n6	cis-13,16-docosadienoic acid	-	-	-	-	-	-	-
C22:6n3	Cervonic acid (DHA)	-	-	-	-	-	33.83 ± 8.02	
Fatty acids index								
%SFA		96.11	90.31	81.61	92.39	81.87	78.26	94.04
%MUFA		2.87	6.29	6.23	5.84	12.32	10.99	1.82
%PUFA		1.02	3.40	12.15	1.77	5.80	10.75	4.14
ω6/ω3		-	1.10	1.07	1.03	1.26	0.89	1.00
IA		10.64	6.41	2.69	7.56	2.58	3.11	5.57
IT		20.76	11.60	3.04	15.22	5.78	3.18	7.05
HH		0.04	0.12	0.27	0.09	0.30	0.25	0.12

Mean and standard deviation of fatty acid data. Lipid quality indices like ω6/ω3 ratio, the atherogenic index (IA), thrombogenic index (IT), and hypocholesterolemic/hypercholesterolemic (HH) index. Data are presented as mean ± standard deviation (SD) of three independent replicates (*n* = 3). Different lowercase letters indicate statistically significant differences among samples (*p* < 0.05).

The index of atherogenicity (IA) represents the atherogenic potential of FAs, as previously described (32). The calculating of IA was following Equation 4:

$$\mathrm{IA}=\frac{C12:0+(4\times C14:0)+C16:0}{\sum \mathrm{UFA}}$$ (4)

Where the IA indicates the relationship between the sum of SFAs and the sum of unsaturated fatty acids (UFAs). The main classes of SFAs, which include C12:0, C14:0, and C16:0, with the exception of C18:0, are considered pro-atherogenic.

The index of thrombogenicity (IT) was also proposed in the same study (32), which was calculated by Equation 5:

$$\mathrm{IT}=\frac{C14:0+C16:0+C18:0}{\begin{array}{l} \left(0.5\times \sum \text{MUFA})+(0.5\times \sum \omega -6\;\text{PUFA}\right)\\ +(3\times \sum \omega -3\;\text{PUFA})+\frac{\omega -3}{\omega -6} \end{array}}$$ (5)

Where the IT characterizes the thrombogenic potential of FAs, indicating the tendency to form clots in blood vessels and provides the contribution of different FAs, which denotes the relationship between the pro-thrombogenic FAs (C12:0, C14:0, and C16:0) and the anti-thrombogenic FAs (MUFAs and the ω-3 and ω-6 families).

The hypocholesterolemic/hypercholesterolemic (HH) ratio was also calculated according to (32), and is used to evaluate the effect of fatty acid composition on cholesterol, which was calculated according to Equation 6:

$$\mathrm{HH}=\frac{\mathrm{cis}-C18:1+\sum \text{PUFA}}{C12:0+(4\times C14:0)+C16:0}$$ (6)

#### 2.3.6 Total carbohydrate content

Total carbohydrate content was estimated by difference from the proximate composition of the dried macroalgal biomass. The calculation was performed as follows Equation (7):

$$\text{Carbohydrate}\;(\%)=100-(\begin{array}{l} \%\text{moisture}+\%\mathrm{ash}\\ +\%\text{lipid}+\%\text{protein} \end{array})$$ (7)

#### 2.3.7 Mineral composition

The contents of macro-elements, including calcium (Ca), potassium (K), magnesium (Mg) and sodium (Na), and microelements, including iron (Fe), manganese (Mn), copper (Cu), chromium (Cr), cobalt (Co), and zinc (Zn), were determinate in the 7 biomasses by inductively coupled plasma optical emission spectrometry (ICP-OES) (33). Prior to analysis, samples were digested with 65% HNO₃ and 30% H₂O₂ in a Multiwave 3,000 (Anton Paar, Graz, Austria). The digestion temperature was increased to 140 °C, 10 min and subsequently maintained at 140 °C for 80 min. After digestion, the solutions were diluted to a final volume of 50 mL with Milli-Q water. Subsequently, the digests samples were analyzed using an ICP-OES instrument (iCAP Pro XP, Thermo Fisher Scientific, Waltham, MA, USA) equipped with a charge injection device (CID) array detector, axial and radial viewing systems, a high dynamic range detector, and a cross-flow nebulizer with mass flow controller (MFC) gas control. Calibration was performed using ICP Calibration Standard 4 and ICP Multielement Standard Solution VI, both prepared in a 2% HNO₃ solution.

#### 2.3.8 Salt content

The content of sodium chloride (NaCl) was estimated by incineration at 600 °C for 5 h. Then, 0.1 g of ash was transferred to a test tube containing 1 mL of nitric acid and 1 mL of hydrogen peroxide and digested at 100 °C for 2 h. The digested solution was analyzed using inductively coupled plasma optical emission spectrometry (ICP-OES) to determine the sodium concentration. Assuming that sodium in the macroalgae samples was presented solely as inorganic NaCl, the NaCl content was estimated based on its chemical formula and molecular weight (33).

### 2.4 Phenolics compounds analysis

#### 2.4.1 Extraction of polyphenolic compounds

Phenolic compounds in macroalgae were extracted using an organic-solvent extraction procedure adapted from Ringgit (34). The powdered samples were mixed with 100% MeOH (1:5, w/v), ultrasonicated at 50 °C for 20 min and subsequently stored overnight at 4 °C. The samples were then centrifuged at 7500 rpm for 5 min, and the supernatant was collected. Then 5 mL of acetone was added to the precipitate and centrifuged at 7500 rpm for 5 min. This extraction step was repeated twice. All supernatants were combined and evaporated at 45 °C using a rotary evaporator (R-100, BUCHI Ibérica S. L. U., Barcelona, Spain). The residue was redissolved in 5 mL of methanol. The solution was filtered through a 0.22 μm filter and stored at −20 °C for subsequent analysis of the phenolic profile.

#### 2.4.2 Total phenolic content (TPC)

The total phenolic content was analyzed by the Folin–Ciocalteu method (35). Briefly, 25 μL of sample and 125 μL of Folin–Ciocalteu reagent, diluted with water at a ratio of 1:10 (v/v), were added to a 96-well plate and incubated at room temperature for 3 min. Thereafter, 100 μL of sodium carbonate solution (75 g/L) was added, and the mixture was homogenized in a plate shaker and incubated at 37 °C for 2 h. Absorbance was measured at 765 nm using a BioTek Synergy HTX Multimode Reader (Agilent, Technologies, Germany). Distilled water was used as a reference. Gallic acid was used as a standard substance to calculate the polyphenol content per 100 g of dry biomass in the extract.

### 2.5 Cytotoxicity of macroalgae extracts

#### 2.5.1 Samples preparation

Macroalgal extracts for cytotoxicity assays were diluted in 50% EtOH with 0.1% formic acid (1:15 w/v) and vortexed, then the mixtures were ultrasonicated in a water bath at 30 °C for 10 min. The samples were shaken at 160 rpm for 30 min and lastly centrifuged at 7500 rpm for 7 min. The supernatant was filtered through a 0.22 μm Nylon membrane before cell viability assays. Total phenolic content was measured as described in section 2.4.2. The cell viability assays for macroalgae samples were based on their total phenolic compound concentration.

#### 2.5.2 Cell culture

HepG2 and Caco-2 cells (Gibco, Thermo Fisher Scientific, Waltham, MA, USA) were cultured in minimum essential medium (MEM) supplemented with 10% fetal bovine serum (FBS), 1% non-essential amino acids (NEAA, 100×) and 1% GlutaMAX™ (100×). The HepG2 culture medium was additionally supplemented with 1% penicillin–streptomycin, while the Caco-2 culture medium contained 0.5% penicillin–streptomycin. Cells were maintained at 37 °C in a humidified atmosphere containing 5% CO_2_. All reagents used for HepG2 and Caco-2 cells culture were purchased from Gibco (Thermo Fisher Scientific, Waltham, MA, USA). HepG2 and Caco-2 cells were typically maintained by changing the culture medium every two days and were used between passages 20 and 30 for cell viability assays (36).

#### 2.5.3 Cell viability assay by Alamar blue assay

HepG2 and Caco-2 cells were seeded in 96-well plates at a density of 40,000 cells per well and incubated for 24 h to allow cell attachment. Macroalgal extracts were freshly prepared by serial dilutions in culture medium prior to treatment. A dose–response design was applied, and the final working concentrations were individually adjusted according to the total phenolic content of each sample to standardize exposure levels and enable accurate comparison of biological effects. After 20 h of treatment with the extracts, Alamar blue reagent was added to each well at a final concentration of 10% (v/v). After an additional 4 h of incubation, cell viability was assessed by measuring absorbance at 570 and 600 nm using a BioTek Synergy HTX Multimode microplate reader (BioTek Instruments, Inc., a part of Agilent Technologies, Winooski, VT, USA). Zinc chloride (ZnCl_2_) at 500 μg/mL was used as the positive control to verify cytotoxic response. All treatments were performed at least in triplicate (36).

#### 2.5.4 Cell viability assay by neutral red assay

The neutral red assay was conducted following the protocol described by Repetto (37). HepG2 and Caco-2 Cells were seeded under the same conditions as those used for the Alamar Blue assay. To avoid interference from Fetal Bovine Serum (FBS), treatments were freshly prepared in HepG2 medium containing 1% FBS. After 24 h of treatment, the culture medium was removed, and the wells were gently washed with PBS.

Neutral red solution (40 μg/mL) was then added to each well, and the cells were incubated for 2 h to allow dye uptake. Subsequently, the wells were washed once with PBS and destained using a solution of 1% glacial acetic acid, 50% EtOH, and 50% water. The absorbance was measured at 540 nm using a BioTek Synergy HTX multimode microplate reader to assess cell viability. All treatments were performed at least in triplicate.

### 2.6 Statistical analysis

All experiments were performed in triplicate, and results are presented as mean ± standard deviation (SD). Statistical analyses were carried out using SPSS 26.0 software (SPSS Inc., Chicago, IL, USA). For each measured variable, including proximate composition, C/N ratio, amino acid profile, fatty acid composition, nutritional indices, mineral content, phenolic content, heavy metal levels and cell viability, differences among macroalgal biomasses were evaluated independently.

Differences among groups were evaluated by one-way analysis of variance (ANOVA), followed by Duncan’s multiple range test for *post hoc* comparison. Statistical significance was accepted at *p* < 0.05. Figures were generated using OriginPro 2024 (version SR1 10.1.0.178, 64-bit) and GraphPad Prism (GraphPad Software, La Jolla, CA, USA).

## 3 Results and discussion

### 3.1 Nutritional composition

The nutritional composition of the 7 macroalgal biomasses, including ash, protein, lipid and carbohydrate contents is presented in Table 3 and represented in Figure 2. Overall, carbohydrates and ash were the predominant fractions in most samples, although marked differences were observed depending on the taxonomic group and biomass origin. Brown macroalgae, namely *S. latissima*, *C. usneoides* and *R. okamurae,* together with the green alga *U. rigida*, generally showed higher ash contents (26 to 52%), while the red macroalgae *P. umbilicalis*, *G. gracilis and the G. corneum* by-products showed higher protein content.

**Table 3:** Nutritional and elemental composition of macroalgae (g/100 g).

Nutritional composition (%)	*U. rigida*	*S. latissima*	*P. umbilicalis*	*G. corneum*	*C. usneoides*	*R. okamurae*	*G. gracilis*
Ash	26.42 ± 0.23^d^	32.05 ± 0.73^c^	9.57 ± 0.31^f^	27.73 ± 2.05^d^	51.88 ± 0.18^a^	38.60 ± 0.31^b^	11.73 ± 0.61^e^
Proteins	9.27 ± 0.30^e^	9.63 ± 0.75d^e^	30.78 ± 0.22^a^	16.48 ± 0.99^c^	9.38 ± 1.54^f^	11.21 ± 0.04^d^	22.69 ± 1.38^b^
Lipids	4.90 ± 0.16^b^	4.31 ± 0.26^c^	4.18 ± 0.09^c^	1.78 ± 0.09^e^	2.78 ± 0.19^d^	4.39 ± 0.17^c^	6.31 ± 0.33^a^
Carbohydrates	59.41 ± 0.69^a^	54.01 ± 1.74^b^	55.47 ± 0.63^b^	54.02 ± 3.12^b^	36.96 ± 1.91^d^	45.80 ± 0.53^c^	59.27 ± 2.32^a^
Elemental composition (%)							
N	2.25 ± 0.07^e^	2.34 ± 0.18^de^	7.47 ± 0.05^a^	4.00 ± 0.24^c^	1.82 ± 0.45^f^	2.72 ± 0.01^d^	5.51 ± 0.33^b^
C	24.19 ± 0.24^de^	26.77 ± 0.03^cd^	40.09 ± 0.17^a^	32.75 ± 2.25^b^	15.53 ± 2.84^e^	30.66 ± 0.08^bc^	32.85 ± 0.58^b^
C/N ratio	10.75	11.44	5.37	8.19	8.52	11.27	5.96

Ash, proteins, lipids and carbohydrates content (% of dry weight) in the macroalgae utilized in the following experiments. Data are presented as mean ± standard deviation, *n* = 3. Different lowercase letters indicate statistically significant differences among samples (*p* < 0.05).

**Figure 2:**
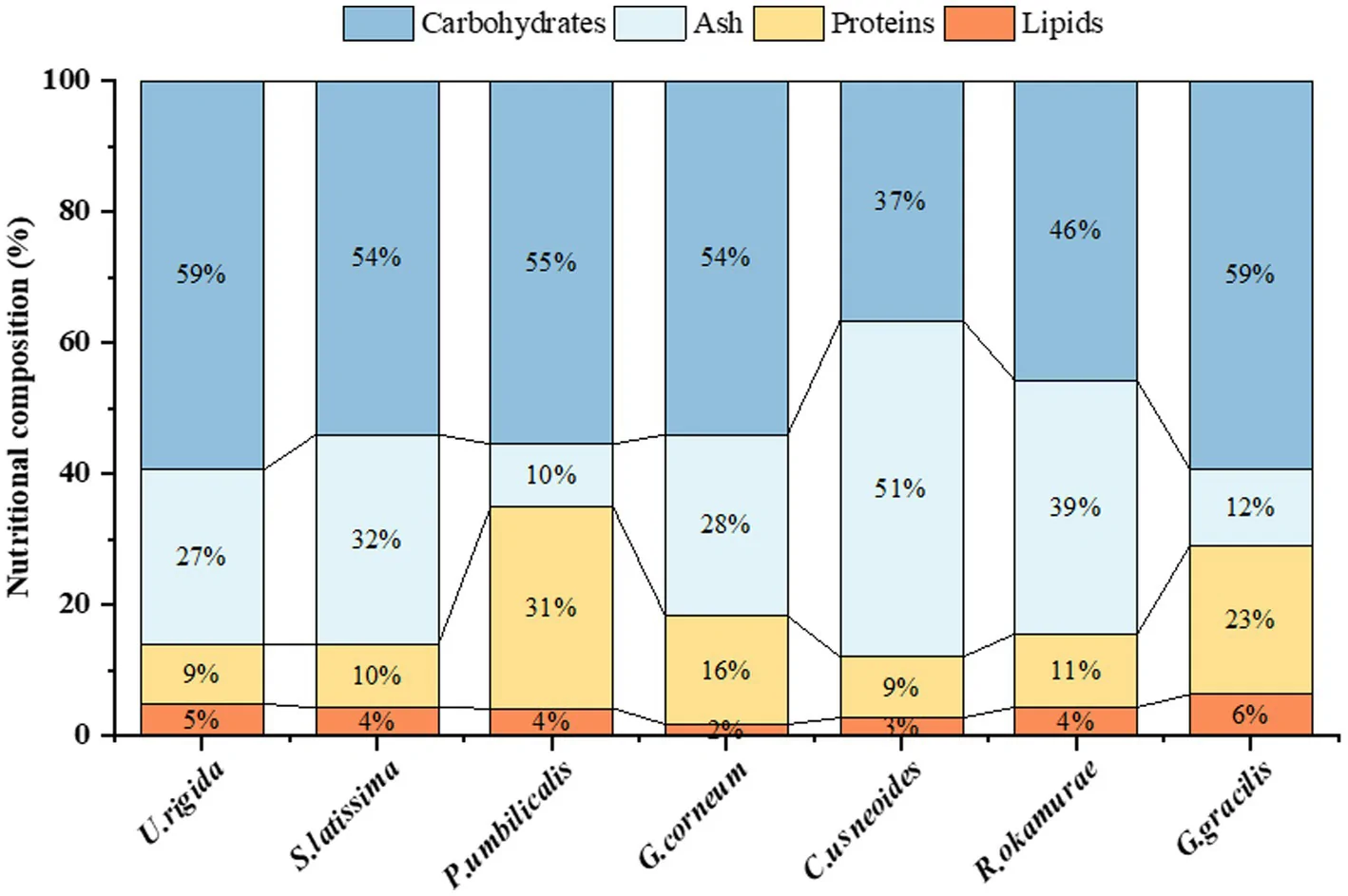
Nutritional composition of macroalgae including the lipid, protein, ash, and carbohydrate content.

It is known that red and green seaweeds generally have higher protein levels than brown seaweeds (38). In agreement with this trend *P. umbilicalis* contains the highest protein level among the evaluated biomasses (31%), followed by *G. gracilis* and *G. corneum* by-product (23 and 16%, respectively). The protein content of *P. umbilicalis* was higher than that previously reported for *Porphyra* spp. collected from the northwestern Iberian coast (24%) (39), but close to the range reported by the Irish edible *P. umbilicalis* (32%) (40). In contrast, brown seaweeds such as *S. latissima*, *C. usneoides* and *R. okamurae* had relatively low protein levels (approximately 9–12%), in agreement with a recent study (41).

Carbon, nitrogen, and the carbon-nitrogen ratio (C/N) ratio are key parameters for assessing the potential suitability of biomass as a substrate for bioprocessing. Carbon provides the energy required for the growth and metabolism of microorganisms, while nitrogen supports protein synthesis, cell proliferation and enzyme production (42). Among the studied samples, *P. umbilicalis and G. gracilis* have the highest nitrogen content (7 and 6%, respectively), together with high carbon contents (40 and 33%, respectively), and the lowest C: N ratio (5.37 and 5.96), indicating a rich nitrogen content. These values suggest that *P. umbilicalis* and *G. gracilis* could be promising nitrogen-rich source for future fermentation-based applications (43). In this regard, Karray (44) reported that during solid-state fermentation of a mixture of *U. rigida* and wheat bran using *Aspergillus niger* CTM 10099, a C/N of 5 favored β -glucosidase activity, while an excessively high C/N ratio would inhibit the enzyme activity. Therefore, the low C/N ratios observed for *P. umbilicalis and G. gracilis* support their potential suitability for bioprocessing. Besides, *S. latissima* and *R. okamurae* also showed the highest C/N ratios, both approximately 11:1, reflecting their comparatively lower nitrogen availability. Compared to terrestrial plants, the protein content of macroalgae is considerably greater, and these proteins are often accompanied by bioactive compounds with antioxidant, antihypertensive, immunomodulatory, anticoagulant, and hepatoprotective properties (45).

Regarding carbohydrates, the content ranged from 37 to 59% of dry biomass. *G. gracilis* and *U. rigida* exhibited the most elevated levels (59.27 and 59.41%, respectively). These results are slightly different from the previous research data. For instance, in the study by Ferreira et al., the carbohydrates contents of *G. gracilis* and *U. rigida* were 53 and 46%, respectively. In the 2015 study by Rodrigues et al., the carbohydrate content of *G. gracilis* was 47% (10, 46). The carbohydrate content of *C. usneoides* was among the lowest of the evaluated biomasses, approximately 37%. Nevertheless, this value is consistent with the reported range of crude polysaccharides or total carbohydrates (approximately 20–48%) for several species of the *Cystoseira* genus, indicating that this species is an important polysaccharide resource (47).

Ash content, indicative of mineral and inorganic element levels of the biomass, was also substantial across all samples, ranging from 8 to 40%, due to the natural tendency of seaweed to accumulate minerals and trace elements from seawater (48). The ash content of *C. usneoides* was the greatest, around 52%, indicating a particularly high content of inorganic elements and minerals. A moderate ash content was observed in brown seaweeds like *S. latissima* and *R. okamurae*, which confirms the trend of higher mineral accumulation in brown algae (49). In contrast, *P. umbilicalis* and *G. gracilis* showed the least ash content, which is consistent with their comparatively higher organic fraction.

Finally, the lipid content was generally low across all macroalgae samples, which is in line with the typical characteristics for this group. A slight exception was *G. gracilis*, which had a slightly elevated lipid level. In all other samples, the lipid content ranged from 1 to 4%.

### 3.2 Amino acid profile and protein quality indices

The nutritional quality of a protein is largely determined by its amino acid composition, particularly the content and balance of essential amino acids. Therefore, the amino acid profiles (AAs) of the 6 macroalgae species and the *G. corneum* by-product were determined through acid hydrolysis to comprehensively understand their potential benefits and their ability as substrates for future bioprocessing applications. The results are shown in Table 4.

**Table 4:** Amino acid profile of macroalgae.

g AA/100 g protein	*U. rigida*	*S. latissima*	*P. umbilicalis*	*G. corneum*	*C. usneoides*	*R. okamurae*	*G. gracilis*
∑EAA	37.58 ± 0.54^bc^	35.68 ± 0.16^d^	34.34 ± 0.52^e^	44.79 ± 0.47^a^	38.46 ± 0.55^b^	31.97 ± 0.36^f^	36.55 ± 1.48^cd^
His	1.42 ± 0.06^e^	1.54 ± 0.04^d^	1.63 ± 0.02^c^	2.51 ± 0.04^a^	2.02 ± 0.03^b^	1.42 ± 0.03^e^	1.25 ± 0.09^f^
Ile	5.69 ± 0.17^c^	5.75 ± 0.06^c^	5.15 ± 0.16^d^	6.97 ± 0.57^a^	6.28 ± 0.30^b^	6.29 ± 0.09^b^	4.14 ± 0.15^e^
Leu	8.18 ± 0.14^bcd^	8.24 ± 0.17^bc^	7.98 ± 0.25^cd^	7.80 ± 0.16^d^	8.57 ± 0.25^b^	8.27 ± 0.18^bc^	9.14 ± 0.33^a^
Lys	4.42 ± 0.04^d^	4.27 ± 0.07^d^	5.92 ± 0.08^b^	9.05 ± 0.17^a^	4.92 ± 0.10^c^	1.18 ± 0.09^e^	4.79 ± 0.48^c^
Met	0.81 ± 0.06^d^	2.37 ± 0.13^a^	0.23 ± 0.16^f^	0.55 ± 0.04^e^	1.16 ± 0.12^c^	0.19 ± 0.14^f^	1.41 ± 0.07^b^
Phe	6.87 ± 0.15^b^	5.55 ± 0.07^e^	5.01 ± 0.10^f^	7.62 ± 0.03^a^	6.31 ± 0.09^c^	5.91 ± 0.02^d^	5.12 ± 0.21^f^
Thr	5.31 ± 0.07^a^	4.19 ± 0.06^c^	3.73 ± 0.12^d^	5.57 ± 0.07^a^	4.61 ± 0.05^b^	4.31 ± 0.07^c^	5.56 ± 0.39^a^
Val	4.87 ± 0.23^b^	3.78 ± 0.04^c^	4.70 ± 0.11^b^	4.73 ± 0.09^b^	4.60 ± 0.08^b^	3.78 ± 0.13^c^	5.13 ± 0.24^a^
∑n-EAA	62.42 ± 0.54^cd^	64.32 ± 0.16^bc^	65.66 ± 0.52^ab^	55.20 ± 0.47^e^	61.54 ± 0.55^d^	68.02 ± 0.36^a^	63.44 ± 3.70^bcd^
Ala	10.66 ± 0.58^c^	13.78 ± 0.50^b^	16.66 ± 0.60^a^	6.80 ± 0.22^e^	8.43 ± 0.18^d^	7.05 ± 0.14^e^	7.91 ± 0.45^d^
Arg	4.85 ± 0.08^c^	3.61 ± 0.06^d^	6.66 ± 0.40^a^	6.06 ± 0.04^b^	5.13 ± 0.07^c^	4.76 ± 0.15^c^	7.02 ± 0.78^a^
Asp	13.11 ± 0.09^a^	11.52 ± 0.15^c^	9.36 ± 0.01^d^	11.81 ± 0.17^bc^	12.20 ± 0.35^b^	11.86 ± 0.21^bc^	12.30 ± 0.56^b^
Cys	0.12 ± 0.02^c^	0.06 ± 0.02^c^	0.59 ± 0.01^b^	0.12 ± 0.02^c^	0.05 ± 0.01^c^	0.07 ± 0.02^c^	1.85 ± 0.17^a^
Glu	11.75 ± 0.31^e^	16.01 ± 0.19^b^	12.70 ± 0.16^d^	9.93 ± 0.10^f^	14.08 ± 0.34^c^	24.15 ± 0.48^a^	13.49 ± 0.80^c^
Gly	7.16 ± 0.34^a^	5.99 ± 0.19^cd^	6.32 ± 0.22^bc^	6.76 ± 0.19^ab^	7.20 ± 0.45^a^	6.82 ± 0.58^ab^	5.47 ± 0.23^d^
Pro	5.57 ± 0.06^a^	5.24 ± 0.16^bc^	4.73 ± 0.11^d^	5.72 ± 0.09^a^	5.11 ± 0.12^c^	4.76 ± 0.11^d^	5.48 ± 0.26^ab^
Ser	5.79 ± 0.04^b^	5.02 ± 0.23^d^	4.49 ± 0.03^e^	4.49 ± 0.05^e^	5.42 ± 0.08^bc^	5.09 ± 0.15^cd^	6.51 ± 0.48^a^
Tyr	3.40 ± 0.04^ab^	3.08 ± 0.16^b^	4.16 ± 0.10^a^	3.49 ± 0.22^ab^	3.92 ± 0.41^ab^	3.44 ± 0.28^ab^	3.43 ± 0.11^ab^
CS							
His	95	102	108	167	135	95	84
Ile	190	192	172	232	209	210	138
Leu	139	140	135	132	145	140	155
Lys	98	95	131	201	109	40	107
Met+Cys	37	108	10	25	53	9	64
Phe+Tyr	181	146	132	200	166	156	135
Thr	231	182	162	242	200	187	242
Val	125	97	121	121	118	97	132
EAAI (%)	121	128	98	141	132	85	123
PDCAAS (%)	29	76	8	20	42	7	51

Amounts expressed in g AA/100 g of protein. Amino acids are represented by the 3-letter abbreviation code. Data are presented as mean ± standard deviation, *n* = 3. AA, amino acids; EAA, total essential amino acids; n-EAA, total nonessential amino acids; CS, chemical score; EAAI, essential amino acid index; PDCAAS, protein digestibility-corrected amino acid score. Different lowercase letters indicate statistically significant differences among samples (*p* < 0.05).

Overall, all samples presented a complete profile of AAs, although the relative contents of individual amino acids varied markedly among species. The *G. corneum* by-product provided the most elevated density of EAAs, containing approximately 44.8 g/100 g protein. *C. usneoides* and *G. gracilis* also showed high EAAs contents, followed by *P. umbilicalis* and *S. latissima* that had similar levels (approximately 38–34 g/100 g protein). Conversely, the proportion of *R. okamurae* was mostly reduced, at only 31.97 g/100 g protein. The total EAA content of *R. okamurae* was comparable to previous reports for this species, with Leu, Phe, and Val among the predominant EAAs (41). Since EAAs cannot be synthesized by the human body and must be obtained from the diet, their relative abundance is a key indicator of protein quality. The abundant EAA levels observed in several samples, particularly in the *G. corneum* by-product, support their potential as valuable dietary protein sources.

Among individual EAAs, His, Ile, Lys, and Phe were markedly higher in *G. corneum* by-product than in the other biomasses. Lys and Phe reached particularly elevated levels in this sample (9.1 g and 7.6 g/100 g protein, respectively). Lys content across other samples was relatively similar, with the exception of *R. okamurae*, which showed a notably lower value. In addition, *S. latissima* exhibited the maximal content of Met among all samples, which is particularly relevant due to sulfur-containing amino acids are involved in key metabolic processes, including methyl-group transfer and antioxidant defense through Glu synthesis (28). It can be observed that among all samples, Leu and Phe appeared to be the most consistently abundant across species. Phe, an aromatic EAA, can be converted into Tyr and subsequently used in the synthesis of neurotransmitters like dopamine and adrenaline and other biologically relevant compounds (50). Its contents in all samples ranged from 5.0 to 7.6 g amino acid per 100 g protein, significantly exceeding the FAO/WHO reference value for adults. In addition, *G. gracilis* showed the highest leucine and valine contents, reaching 9.1 g/100 g protein and 5.1 g/100 g protein, respectively. These branched-chain amino acids may be inclined toward energy metabolism (such as ethanol) and the generation of short-chain organic acids in microbial metabolism, thereby affecting the distribution of fermentation products (51).

Regarding non-essential amino acids (n-EAA), Asp., Ala, and Glu were the most representative components, consistent with previous studies on macroalgal proteins (52). *R. okamurae* showed the highest level of n-EAA content (about 68 g/100 g protein), with Glu comprising a substantial proportion (24.2%). This may be relevant for microbial nitrogen metabolism and the synthesis of intermediate metabolites during bioprocessing (53). Meanwhile, *P. umbilicalis* presented the highest Ala content, 16.7 g/100 g protein. Studies show that higher levels of alanine can serve as a potential carbon source to increase lactic acid yield through transamination and reduction pathways (54). Although *G. gracilis* had a relatively high Cys content (1.9 g / 100 g protein), the overall Cys levels of all samples were low, which might be related to the differences in amino acid extraction methodologies (24). On the other hand, the contents of Gly, Pro, Ser, and Tyr were relatively consistent across all macroalgae, showing no significant interspecies differences.

To evaluate the nutritional quality of EAA compounds in the samples, three protein quality indices were calculated: CS, EAAI, and PDCAAS. CS is a fundamental indicator used to evaluate whether the content of each EAA in the protein meets human nutrition requirements. As shown in Table 4, Met + Cys was identified as the limiting amino acid in most samples, while Lys was the limiting amino acid in *S. latissima*. Based on the FAO/WHO/UNU recommended adult amino acid pattern (27), EAAI values of all samples were calculated to estimate the overall balance of essential amino acids (55). The *G. corneum* by-product showed the highest EAAI value (141%), while the others ranged from 132 to 85%. These differences may be attributed to factors such as species, geographical origin, harvest season, and environmental conditions (56). High-quality and efficient proteins typically have best EAAI values, an EAAI above 70% indicates medium to high protein quality (33). This suggests that these seaweed species may serve as valuable sources of high-quality protein. The high EAA content and EAAI of the *G. corneum* by-product are consistent with previous reports showing that agar-extraction residues retain nutritionally relevant proteins and amino acids that can be further recovered for protein valorization (57). In addition, *G. corneum* residues have been evaluated as substrates for solid-state fermentation with *Aspergillus* spp., indicating their feasibility for further bioprocessing (58).

Moreover, protein quality evaluation must also consider digestibility, which more accurately reflects the protein’s utilization in the human body. The *in vitro* digestibility of seaweed proteins was relatively similar across species, averaging around 80% (29). However, in this study, PDCAAS values among the analyzed samples varied widely, ranging from 7 to 76%, with *S. latissima* and *G. gracilis* exhibiting the greatest PDCAAS. These differences are likely due to the structural characteristics of seaweed cell walls, which are rich in cellulose and polysaccharides, often entrapping amino acids and reducing their bioavailability compared to sources like soy protein (59). On the other hand, the EAAI value of *P. umbilicalis* and *G. gracilis* were higher than 70%, indicating that its amino acid composition was relatively balanced but not optimal, but its PDCAAS value is only 8%, far lower than that of other algae, indicating that the digestibility of the protein is restricted. This characteristic may be attributed to the fact that the cell walls of red algae are rich in polysaccharides such as agarose and galactomannan, and proteins are often embedded or cross-linked with polyphenols, which hinders their digestion and absorption (60). From a bioprocessing perspective, this low-release effect may serve as a “slow-release nitrogen source”, which helps maintain protease induction and continuous growth/metabolism for a longer period of time. Previous studies have shown that the use of *P. umbilicalis* hydrolysate for Lactic acid bacteria (LAB) or yeast fermentation can enhance protein utilization and biological activity, which further supports its potential as a fermentation substrate (61).

Overall, the studied macroalgal biomasses were characterized by relevant levels of EAA and abundant acidic amino acids, particularly aspartic and glutamic acid. Solid-state fermentation has been reported to improve protein accessibility and digestibility in plant and macroalgal substrates, although the effect depends on the substrate and microbial strain (62). The relatively high protein and EAA contents of *P. umbilicalis*, *G. gracilis*, and the *G. corneum* by-product therefore support their further evaluation as fermentation feedstocks. These compounds may serve as important nitrogen sources for microbial nitrogen metabolism and may contribute to microbial growth and biomass accumulation during fermentation. Therefore, the amino acid profiles of the evaluated macroalgae not only affect their protein nutritional value, but also determine their metabolic responses and product patterns when used as fermentation substrates.

### 3.3 Fatty acids profile and lipid quality indices

The fatty acid profiles of the six macroalgal species and the *G. corneum* by-product are presented in Table 2. Total fatty acid content varied markedly among samples, with *G. gracilis* showing the lowest value (314 mg/kg). SFAs were the predominant lipid fraction in all biomasses, with proportions ranging from 78.26% (*R. okamurae*) to 96.11% (*U. rigida*). Among them, the most abundant SFA are palmitic acid (C16:0) and stearic acid (C18:0), both of which are relatively common in marine organisms (63). *R. okamurae* showed the highest myristic acid (C14:0) content, reaching 469 mg/kg and accounting for 15% of the FA. It has been reported for having potential in reducing inflammation and antioxidation (41). Generally, a high SFA content indicates that the lipids of these algae have strong oxidative stability, but it may have a certain impact on the nutritional structure (64).

The greatest levels of MUFA were observed in *C. usneoides* (778 mg/kg) and *R. okamurae* (766 mg/kg), primarily derived from oleic acid (C18:1n9) and palmitoleic acid (C16:1n7). In contrast, despite the relatively low overall proportion of PUFA, *P. umbilicalis* and *R. okamurae* exhibited the highest PUFA levels, each accounting for approximately 12% of total fatty acids. *P. umbilicalis* was especially noteworthy for its high content of eicosapentaenoic acid (EPA, C20:5n3), an important ω-3 fatty acid with anti-inflammatory and cardioprotective properties, reaching 311 mg/kg (65). *R. okamurae* also demonstrated a considerable EPA content (213 mg/kg DW) and was the only species in which both docosahexaenoic acid (DHA, C22:6n3) and γ-linolenic acid (C18:3n6) were detected, at 33 mg/kg and 23 mg/kg, respectively. These findings highlight the potential of specific macroalgae as valuable sources of marine-derived bioactive fatty acids, particularly ω-3 fatty acids, for use in nutritional and functional food applications (66).

From a bioprocessing perspective, the presence of unsaturated and long-chain fatty acids (such as EPA) may also be relevant, as microorganisms with fatty acid oxidation capacity can convert these compounds into acetyl-CoA and reduced equivalents (NADH, FADH₂) through β -oxidation. This process may provide additional energy for fermentation and help sustain metabolic activity when carbon sources are limited (67). Moreover, fermentation can hydrolyze lipids in the substrate and generate free fatty acids and their oxidized derivatives. These products often have anti-inflammatory or antioxidant activities, thereby enhancing the functionality of the fermentation products (68).

Significantly, the overall ω-6/ω-3 ratio ranged from 0.89 to 1.26 among the evaluated biomasses, reflecting a balance proportion of this PUFA families. This parameter is nutritionally relevant because an appropriate ω-6/ω-3 ratio is essential for maintaining immune regulation, inflammatory response, cardiovascular health, and proper nervous system function (69). To further assess the cardiovascular health potential of the fatty acid profiles, the IA, IT, and the HH were also evaluated. IA and IT serve as indicators of the balance between saturated and UFAs, with lower values suggesting a higher proportion of heart-beneficial UFAs (70). Consuming foods or products with least IA values may help reduce total plasma cholesterol and low-density lipoprotein (LDL) cholesterol levels (71). The HH index is considered a more accurate reflection of the potential impact of fatty acid composition on cardiovascular health. A larger HH value is positively correlated with PUFA content, indicating greater health benefits (32). Among the species analyzed, *P. umbilicalis* and *R. okamurae* exhibited relatively low IA and IT values and larger HH values, indicating a favorable profile of unsaturated fatty acids, particularly ω-3 fatty acids. Paiva (72) reported similar findings, with HH values ranging from 1.26 to 2.09 in four macroalgal species, highlighting the potential of macroalgae as sources of bioactive lipids, although their overall lipid contribution remains limited due to the generally low total lipid content of seaweed biomass.

### 3.4 Mineral composition

From a nutritional and bioprocessing perspective, mineral elements are crucial because they contribute to human nutrition and are also essential for enzyme activity and metabolism during the fermentation process. Therefore, the mineral composition of the six macroalgae and the *G. corneum* by-product was quantitatively analyzed, as shown in Table 5.

**Table 5:** Mineral compositions of macroalgae.

Mineral	*U. rigida*	*S. latissima*	*P. umbilicalis*	*G. corneum*	*C. usneoides*	*R. okamurae*	*G. gracilis*
Macro elements (g/kg)							
Ca	3.98 ± 0.09^d^	11.69 ± 0.26^c^	2.68 ± 0.01^d^	41.88 ± 1.58^a^	32.29 ± 4.41^b^	12.63 ± 1.41^c^	2.40 ± 0.03^d^
K	23.00 ± 0.33^d^	71.66 ± 0.81^a^	27.42 ± 0.12^b^	0.89 ± 0.07^e^	21.16 ± 5.09^d^	53.57 ± 2.89^b^	54.07 ± 1.00^b^
Mg	34.97 ± 0.26^a^	7.75 ± 0.14^bc^	3.11 ± 0.03^d^	3.28 ± 0.18^d^	5.63 ± 1.12^cd^	9.08 ± 3.38^b^	4.12 ± 0.02^d^
Na	31.22 ± 0.36^b^	38.91 ± 0.74^a^	7.09 ± 0.14^f^	1.21 ± 0.02^g^	10.42 ± 2.55^e^	26.78 ± 1.98^c^	15.70 ± 0.05^d^
Na/K	1.36	0.54	0.26	1.37	0.49	0.50	0.29
Micro elements (mg/kg)							
Fe	762.47 ± 53.45^c^	191.13 ± 71.37^c^	159.86 ± 6.93^c^	2658.28 ± 212.33^b^	1231.37 ± 103.20^bc^	2730.08 ± 1928.14^b^	4730.02 ± 62.18^a^
Mn	55.32 ± 0.55^c^	7.85 ± 0.72^d^	23.22 ± 0.19^cd^	109.98 ± 2.12^b^	22.05 ± 11.94^cd^	56.02 ± 30.93^c^	490.06 ± 31.73^a^
Cu	15.08 ± 0.09^a^	0.67 ± 0.07^f^	13.72 ± 0.04^b^	6.05 ± 0.15^c^	3.48 ± 0.51^e^	6.16 ± 0.92^c^	4.80 ± 0.04^d^
Co	0.99 ± 0.03^ab^	0.03 ± 0.03^b^	0.14 ± 0.01^b^	1.69 ± 0.27^ab^	0.52 ± 0.22^b^	2.64 ± 2.19^a^	0.24 ± 0.01^b^
Cr	1.80 ± 0.11^b^	1.32 ± 0.51^b^	0.35 ± 0.03^b^	117.38 ± 80.13^a^	25.33 ± 20.96^b^	31.70 ± 17.47^b^	0.45 ± 0.03^b^
Ni	2.53 ± 0.10^b^	0.64 ± 0.12^b^	0.58 ± 0.04^b^	59.24 ± 39.04^a^	3.10 ± 1.57^b^	18.76 ± 1.60^b^	0.48 ± 0.04^b^
Zn	11.24 ± 0.42^e^	22.21 ± 0.03^d^	31.85 ± 0.19^c^	61.43 ± 2.93^a^	19.01 ± 3.12^d^	13.17 ± 3.27^e^	48.40 ± 0.44^b^
Salt content (mg/kg)							
NaCl	18.98 ± 0.32^a^	19.69 ± 0.31^a^	10.87 ± 0.28^b^	0.62 ± 0.34^b^	3.57 ± 0.26^c^	10.25 ± 0.25^d^	1.86 ± 0.30^e^

NaCl equivalent estimated from sodium concentration, mg/kg DW. Data are presented as mean ± standard deviation (SD) of three independent replicates (*n* = 3). Different lowercase letters indicate statistically significant differences among samples (*p* < 0.05).

The mineral composition varied significantly among the evaluated samples, showing unique nutritional characteristics. Overall, the samples were rich in macronutrients such as Ca, K, Mg, and Na, in agreement with previous reports on macroalgal biomass (73). For instance, the one with the highest Ca content is *G. corneum* by-product (41.88 g/kg), suggesting that this industrial side stream may retain a relevant mineral fraction. Brown macroalgae tend to accumulate a relatively large amount of K and Na, like *S. latissima* and *R. okamurae,* which exhibited Na contents of 38.91 and 26.78 g/kg, respectively, and K contents of 71.66 and 53.57 g/kg, respectively (10, 74). According to the daily intake (RDA) recommended by the EU and the WHO, together with the reported average daily of 8 g of dried seaweed in the Asian diet (75), the evaluated macroalgae may contribute differently to mineral intake depending on their species-specific composition. Among them, brown algae are rich in Na and K and can serve as a good alternative source of salt in food. The red algae *Phymatolithon calcareum* is the main contributor to the RDA of *Ca.* Additionally, Mg was found to be particularly abundant in green macroalgae, which is consistent with the result that *U. rigida* had an extremely high Mg content (34.97 g/kg) (5).

Regarding Fe, Mn and Zn, *G. gracilis* and *G. corneum* by-product have the highest Fe content (4,730 and 2,658 mg/kg, respectively). These biomasses also showed relevant Mn contents (490 and 109 mg/kg, respectively) and Zn contents (48 and 61 mg/kg, respectively). This agrees with previous studies, showing that red algae tend to accumulate more Mn and other trace elements (5). From a bioprocessing perspective, these minerals are indispensable for maintaining the enzymatic activity and metabolic function of fermentation microorganisms. For instance, Zn is a core component of important metabolic enzymes such as alcohol dehydrogenase, mediating electron transfer and redox reactions, and directly influencing substrate utilization efficiency and product generation (74). Similarly, Ferreira et al. (76) studied the effects of two types of *Aspergillus* on solid-state fermentation of macroalgae and found that higher carbohydrate content (especially cellulose and free sugar) as well as Mg, Mn and Fe was positively correlated with enzyme yield after fermentation.

Overall, the evaluated macroalgal biomasses are naturally rich in metal ions such as Mg, Mn, Zn, and Fe, which can reduce the supplementation of nutrients in fermentation and enhance the metabolic activity of the bacteria. Nevertheless, their application in food or feed systems should be accompanied by safety assessment, especially considering the possible co-accumulation of potentially toxic elements in seaweed biomass.

### 3.5 Salt content

The estimated salt content of the six macroalgal species and the *G. corneum* industrial by-product is shown in Table 5. Salt (NaCl) content of different macroalgae varies greatly, reflecting differences associated with species, habitat, processing conditions and post-harvest treatment. Among them, the NaCl contents of *S. latissima* and *U. rigida* were the most pronounced, reaching 19.69 and 18.98 mg/kg, respectively. These values indicate that they might have a strong salt enrichment ability. In contrast, *G. corneum* by-product and *G. gracilis* have the lowest NaCl contents, with values of 0.62 mg/kg and 1.82 mg/kg, respectively. In the case of *G. corneum* by-product, this lower value might be due to the dissolution or removal of some inorganic salts by algae during the extraction process. On the other hand, brown algae are the main contributors of Na and K, indicating that they can be good candidates as salt substitutes in food formulas. In fact, many studies have found that seaweed can be used as a substitute for NaCl in common foods, while increasing the intake of minerals that are usually insufficient in European populations (6). Nevertheless, the use of macroalgae as salt substitutes should be carefully evaluated, particularly in species with high sodium levels.

In addition, salt content may affect cell growth. Studies have found that the growth of various LAB and yeasts is significantly inhibited at 3–5% (w/v) NaCl, with stronger inhibition usually observed above 5%. However, moderate salinity is suitable for traditional fermentation (such as kimchi and soy sauce), as it can inhibit some pathogenic or spoilage bacteria while allowing salt-tolerant fermentation bacteria (such as certain LAB, yeasts, and *Aspergillus*) to grow, thereby enhancing microbial safety and flavor formation (77). Therefore, the species-specific salt content of macroalga should be considered when selecting biomass for food processing or fermentation-based applications.

### 3.6 Total phenol content (TPC)

Macroalgae are recognized as a good source of phenolic compounds with potential antioxidant properties, which supports their application in the food, feed, cosmetics and pharmaceutical industries. In this study, the TPC of macroalgal samples was determined, and the results are shown in Figure 3.

**Figure 3:**
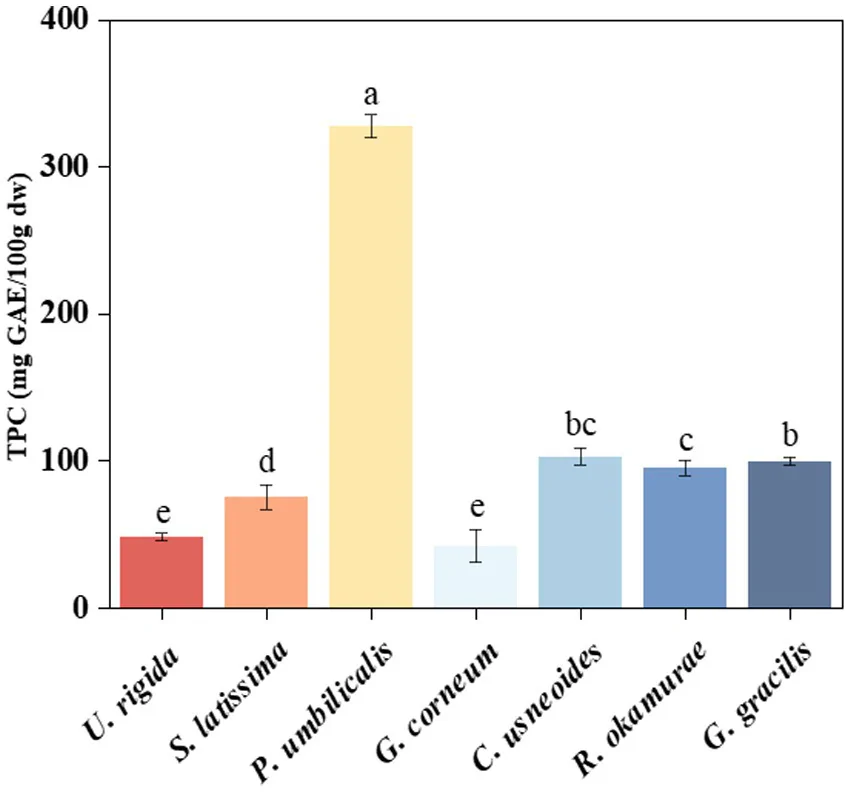
Total phenolic content of the evaluated macroalgal biomasses: *U. rigida*, *S. latissima*, *P. umbilicalis*, *G. corneum* industrial by-product, *C. usneoides*, *R. okamurae* and *G. gracilis*. Results are expressed as mg gallic acid equivalents (GAE)/100 g dry biomass. Data are presented as mean ± standard deviation (SD) of three independent replicates (*n* = 3). Error bars represent SD. Different lowercase letters indicate statistically significant differences among samples (*p* < 0.05).

Significant differences in TPC were observed among the different seaweed species studied. *P. umbilicalis* exhibited the highest TPC (328 mg GAE/100 g dry biomass), exceeding values previously reported in the literature (78). In contrast, the *G. corneum* industrial by-product presented the lowest TPC (43 mg GAE/100 g). *C. usneoides*, *R. okamurae*, and *G. gracilis* showed intermediate phenolic contents, with values around 100 mg GAE/100 g, suggesting their potential antioxidant activity (79).

Studies have shown that brown seaweeds generally contain higher levels of phenolic compounds compared to red and green seaweeds (80). However, in the present study, the highest TPC was observed in the red alga *P. umbilicalis*, highlighting the strong influence of species-specific composition, environmental conditions and extraction methodology on phenolic recovery. Indeed, phenolic compounds in macroalgae are highly dependent on the extraction conditions employed. Ferreira (81) compared three extraction methods (hydroethanolic extraction, water infusion, and water decoction), on the red seaweed *P. umbilicalis*, and found that the water infusion extract exhibited the most elevated TPC (28.38 mg GAE/g), significantly surpassing that obtained by hydroethanolic extraction. Overall, extraction techniques, solvent selection, and solid-to-liquid ratios are critical factors that significantly influence the measured TPC in seaweed samples (82).

From a valorization perspective, phenolic compounds are of interest not only because of their potential antioxidant activity, but also because they may contribute to the development of functional ingredients. Furthermore, numerous studies have shown that fermentation is an effective method to enhance the nutritional value and antioxidant activity of macroalgal biomass. During the fermentation process, enzymes such as amylase, cellulase and other cell wall-degrading enzymes can destroy the cell walls of algae, resulting in the release of free phenolic compounds and flavonoids (83). For example, phenolic compounds and flavonoids were enhanced by fermentation of *Saccharina japonica* powder with *Monascus purpureus* (84). These findings suggest that selected macroalgal biomasses, particularly those with relevant phenolic contents such as *P. umbilicalis*, could be further explored for the production of functional ingredients through fermentation or other bioprocessing strategies.

### 3.7 Heavy metals and iodine

In regions with industrialization and rapid economic development, heavy metal pollution in aquatic systems is becoming increasingly serious. Since macroalgae can accumulate elements from seawater the contents of As, Cd, Pb, and Hg were determined in the evaluated biomasses to assess potential safety-related concerns (Table 6).

**Table 6:** Heavy metals content of samples expressed in mg/kg of dry biomass.

Macroalgae	Cd	Hg	Pb	As	I
*U. rigida*	0.034	0.009	0.784	1.82	207
*S. latissima*	0.275	0.017	0.070	63.20	2041
*P. umbilicalis*	3.401	0.013	0.145	25.20	104
*G. corneum*	0.982	0.010	3.569	2.51	183
*C. usneoides*	0.058	0.072	1.118	189.98	2,270
*R. okamurae*	0.060	0.009	0.769	24.59	4,242
*G. gracilis*	0.026	0.015	1.043	8.02	355

In Europe, seaweed is regarded as a “new type of food”. However, so far, there are no harmonized regulations controlling the maximum levels of potential toxic elements in seaweed. In this context, France internal guidelines are implemented by the French Public Health Council, as reference limits for seaweed consumption, including Cd being 3.0 mg/kg, Hg 0.1 mg/kg, Pb 5 mg/kg, and iAs 3 mg/kg (17). As is one of the main pollutants of concern in macroalgae and usually exists in organic or inorganic forms. In this study, no morphological analysis of As was conducted. Therefore, the share of inorganic forms in the total arsenic pool is unknown. Consequently, total As values should be interpreted with caution and cannot be directly equated to inorganic As exposure.

Overall, the maximal As content was found in brown seaweeds, while red and green seaweeds generally showed lower values, ranging from 1.82 to 25.20 mg/kg. The enrichment of As in macroalgae mainly depends on both intrinsic biological characteristics, such as biomass composition and cell wall structure, and secondly, by the external environmental conditions (85). It has been reported that the As content of *S. latissima* in macroalgae samples collected from various parts of the world is relatively high at 54.4 mg/kg, which is similar to the result of this study (63.2 mg/kg) (86). Nevertheless, 189 mg/kg of As was still observed in *C. usneoides*, which deserves special attention and indicates the need for further As speciation before considering food-related applications. In contrast, *R. okamurae* showed a lower total As content of 24.59 mg/kg, and the contents of Cd, Hg and Pb are relatively low and do not exceed the prescribed limits.

In addition, Cd, Hg and Pb are also metals worthy of attention due to their potential health effects. Cd exposure is mainly associated with kidney and bone damage and long-term exposure can lead to renal failure and osteoporosis. Hg and Pb are extremely harmful to the nervous system, especially methylmercury, which can cause intellectual impairment and abnormal fetal development (87). In the present study, Cd content in *P. umbilicalis* exceeds the reference limit, suggesting that its direct use as a food ingredient should be carefully evaluated. Conversely, Hg and Pb contents of all the samples did not exceed the prescribed limits.

The observed high I levels can be considered as typical for macroalgae. The highest content was reported for species of brown macroalgae, which is consistent with the literature data (88). For example, it was reported that the I content of *S. latissima* collected from the Scottish region was as high as 4,855 mg/kg, whereas the content for green algae such as *Ulva* spp. are generally lower, between 66–130 mg/kg. Red algae such as *Porphyra* spp. usually present comparatively lower I contents, around 40 mg/kg, which is consistent with the results obtained in this study (5). Although iodine is an essential micronutrient, excessive intake may cause thyroid dysfunction, including goiter or hyperthyroidism, particularly when high-iodine seaweeds are consumed regularly (89). However, seaweed is regarded as a new resource food in Europe, and its consumption is still not sufficient to exceed the recommended level (90).

### 3.8 Cell toxicity screening

Because macroalgae are complex matrices with multiple nutrients, bioactive and potentially toxic compounds, cell-based assays can provide a useful preliminary screening tool to assess possible safety concerns. The liver plays a central role in the metabolism of compounds absorbed after digestion, including potentially harmful substances, and may either reduce or enhance the impact of ingested toxins, making it easier for liver cells to respond to metabolites and absorbed toxins (91). Therefore, the cytotoxicity of the macroalgal extracts was first tested using the HepG2 liver cancer cell line. If there are any changes in HepG2 activity caused by macroalgae samples, tests will also be conducted on the Caco-2 colon adenocarcinoma cell line to complement the toxicity screening. Cell viability was evaluated using two complementary methods: Alamar blue and neutral red.

HepG2 cell viability assays revealed lower viability only for *P. umbilicalis*, *R. okamurae*, and *C. usneoides* (Figure 4). The IC_50_ values obtained from the Alamar blue assays were 17.9, 4.3, and 7.4 μg/mL of total phenolic compounds, respectively. On the other hand, the neutral red uptake assay resulted in similar IC_50_ values for *R. okamurae* (4.3 μg/mL), and *C. usneoides* (7.3 μg/mL), but a lower IC_50_ value of 2.1 μg/mL for *P. umbilicalis*. Based on these results, the extracts from these three macroalgae were also evaluated in Caco-2 cell line (Figure 5). *P. umbilicalis* did not negatively affect Caco-2 cell viability under the tested conditions, and therefore no IC_50_ value was calculated. Conversely, *C. usneoides* and *R. okamurae* were able to reduce cell viability using both assays. For *R. okamurae,* IC_50_ values of 4.1 and 3.5 μg/mL were obtained based on neutral red uptake and Alamar blue, respectively. On the other hand, *C. usneoides* treatment resulted in IC_50_ values of 11.7 and 9.2 μg/mL based on neutral red uptake and Alamar blue, respectively.

**Figure 4:**
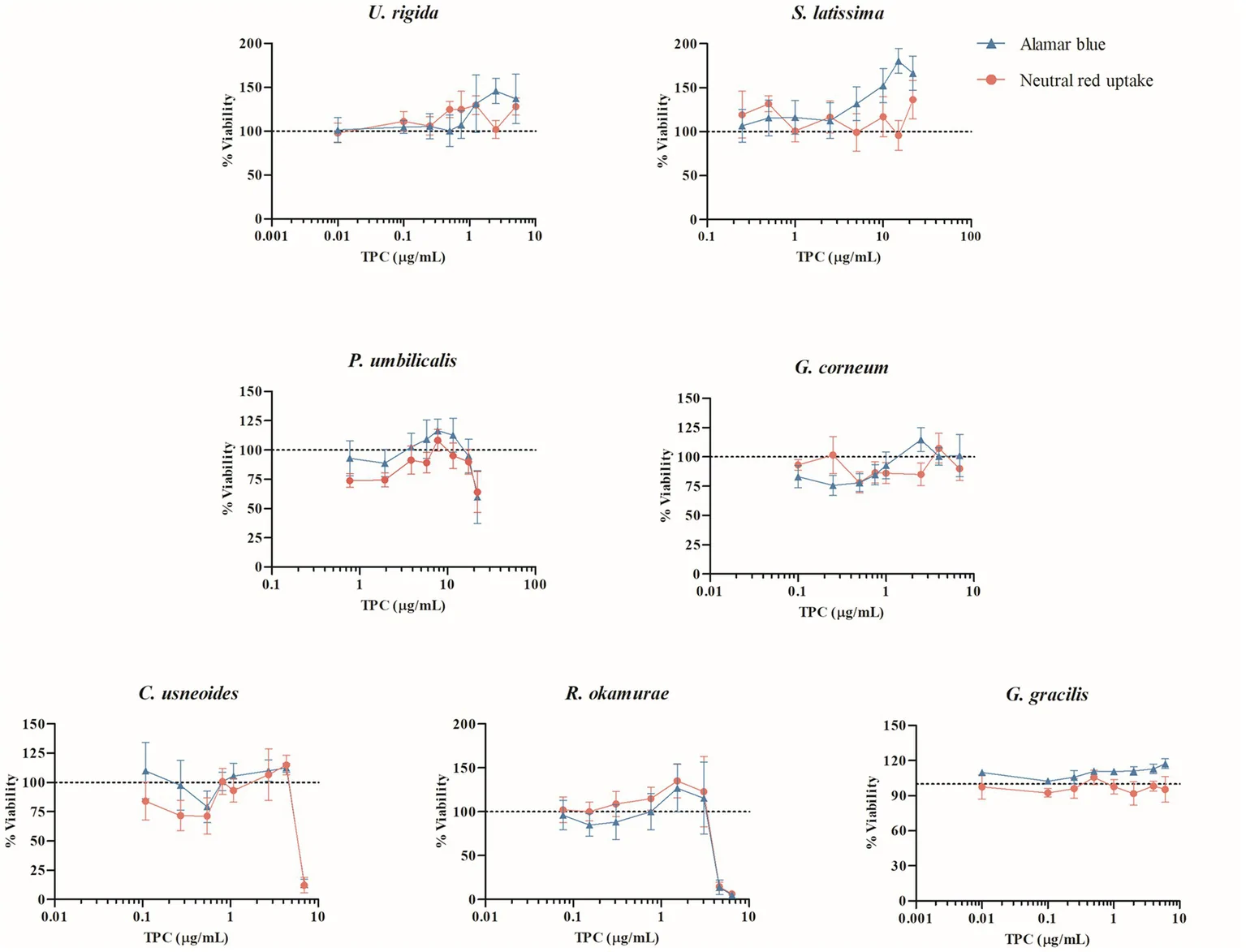
Alamar blue (blue triangles) and neutral red uptake (red circles) cell viability assay in HepG2 cells after 24 h of treatment. The horizontal dotted line indicates 100% viability. Mean ± standard error of the mean are represented, *n* = 3.

**Figure 5:**
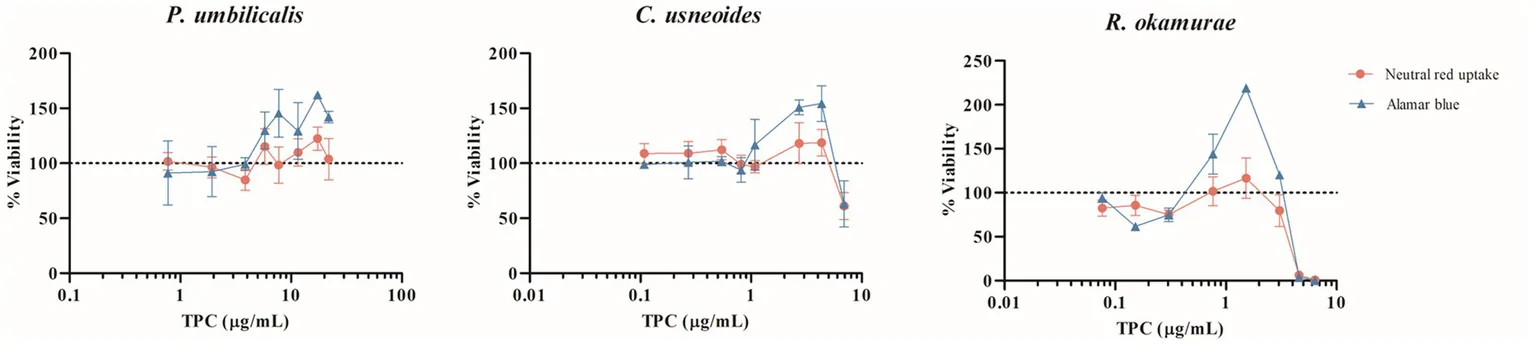
Alamar blue (blue triangles) and neutral red uptake (red circles) cell viability assay in Caco-2 cells after 24 h of treatment for *P. umbilicalis*, *C. usneoides*, and *R. okamurae*. The horizontal dotted line indicates 100% viability. Mean ± standard error of the mean are shown, *n* = 3.

Taken together, the extracts that altered cell viability were those obtained from *P. umbilicalis*, *C. usneoides* and *R. okamurae*. It should be noted that *P. umbilicalis* caused this effect only on HepG2, which may be related, at least in part, to the high concentration of phenolic compounds in this species. In this regard, Ferreira et al. (81) determined the cell viability of different extracts of *P. umbilicalis* using RAW 264.7 cells and found that the content of TPC was significantly and strongly negatively correlated with the cell viability. In the case of *C. usneoides,* the reduction in cell viability may be due to the sample content of arsenic even though the inorganic form was not tested (Table 6). Although *R. okamurae* presented lower concentration of As (Table 6), showed a relatively high Fe content (Table 5), and it has been reported that non-toxic concentrations of Fe potentiate arsenic toxicity. The fact that we are working with extracts and not isolated samples can explain some of the differences in the reduction of cell viability. However, this only happened at considerable levels of the test sample (around 10%), therefore, macroalgae were relatively safe (92).

## 4 Conclusion

This study provides an integrated nutritional and toxicological characterization of seven Atlantic macroalgal biomasses, including the invasive *R. okamurae*, the underutilized *C. usneoides*, and an industrial by-product derived from *G. corneum*. The results highlight the potential of macroalgae as protein-oriented resources, particularly the red macroalgae *P. umbilicalis* and *G. gracilis,* and *G. corneum* by-products, which showed the most promising overall features for alternative protein production, mainly due to their high protein contents, favorable C/N ratios and valuable essential amino acid profiles.

Beyond protein composition, selected biomasses also provided valuable minerals, phenolic compounds and bioactive fatty acids, supporting their possible use within food, feed or biorefinery-oriented applications. In particular, the *G. corneum* by-product represents an interesting circular economy resource, since its previous industrial processing may facilitate nutrient accessibility and further valorization. However, safety-related constraints must be considered, especially the elevated Cd content detected in *P. umbilicalis* and the high As content observed in *C. usneoides*, which may limit their direct use as food ingredients without further assessment.

Overall, *P. umbilicalis*, *G. gracilis* and *G. corneum* by-product emerged as the most suitable candidates for future protein-oriented bioprocessing and functional ingredient development. Future research should integrate biomass structural features, pretreatment process, and fermentation performance to establish a more comprehensive evaluation framework, thereby promoting the application of macroalgae in alternative protein fermentation.

## Data Availability

The original contributions presented in the study are included in the article/supplementary material, further inquiries can be directed to the corresponding authors.
